# Prevalence of benign focal liver lesions: ultrasound investigation of 45,319 hospital patients

**DOI:** 10.1007/s00261-015-0605-7

**Published:** 2016-01-13

**Authors:** Tanja Eva-Maria Kaltenbach, Phillip Engler, Wolfgang Kratzer, Suemeyra Oeztuerk, Thomas Seufferlein, Mark Martin Haenle, Tilmann Graeter

**Affiliations:** Department of Internal Medicine I, University Hospital Ulm, Albert-Einstein-Allee 23, 89081 Ulm, Germany; Department of Interventional and Diagnostic Radiology, University Hospital Ulm, Albert-Einstein-Allee 23, 89081 Ulm, Germany; Zentraler Ultraschall, Klinik für Innere Medizin I, Zentrum für Innere Medizin, Universitätsklinikum Ulm, Albert-Einstein-Allee 23, 89081 Ulm, Germany

**Keywords:** Focal liver lesions, Hepatic cysts, Hepatic hemangioma, Focal nodular hyperplasia, Hepatic adenoma, Focal fatty sparing

## Abstract

**Purpose:**

The aim of the study was to determine the sonographic prevalence of benign focal liver lesions on the basis of a population of hospital patients.

**Methods:**

The ultrasound results in a population of (*n* = 45,319) hospital patients over a period of 10 years were examined retrospectively and evaluated for the diagnosis of benign focal liver lesions [hepatic cysts, hepatic hemangioma, focal nodular hyperplasia (FNH), hepatic adenoma, and focal fatty sparing]. Results that were incomplete or ambiguous were excluded from this study.

**Results:**

At least one of the lesions to be investigated was diagnosed in 15.1% (*n* = 6839) of the patients of the total population. The most commonly recorded lesion, with a total prevalence of 6.3% (*n* = 2839), was focal fatty sparing, followed by hepatic cysts with 5.8% (*n* = 2631). The prevalence of hepatic hemangioma was 3.3% (*n* = 1640), while that of FNH was 0.2% (*n* = 81) and that of hepatic adenoma was 0.04% (*n* = 19). An association between the occurrence of benign focal liver lesions and age was observed.

**Conclusions:**

The calculated prevalence of benign focal liver lesions shows that on the fortuitous discovery of space-occupying lesions of the liver, first consideration should be given to focal fatty sparing, simple hepatic cysts and hemangiomas. The finding of a FNH or an adenoma is rarely a random discovery.

Abdominal ultrasound, particularly of the liver, is a widely available, inexpensive technique that can be rapidly performed without exposing the patient to radiation. It is therefore the method of choice in primary diagnostic investigations in most specialist areas of medicine, but especially for imaging the abdomen [1]. Due to the continuously improving technical standard of ultrasound equipment and the high number of abdominal ultrasound examinations, the number of—often fortuitously—discovered focal liver lesions, the so-called “incidentalomas,” is also increasing markedly [2]. The investigating physician is therefore increasingly faced with the problem of differentiating between malignant and benign space-occupying processes and of distinguishing the various lesions from each other [2–4]. It is important for the subsequent diagnosis and therapy and the associated expended time and effort of the treating physician and the affected patient—to say nothing of the related costs—that the focus visible on ultrasound is classified as reliably as possible [4]. Besides the clinical parameters and the patient’s medical history, the quality of the ultrasound equipment used and the investigator’s experience also play a significant role. 57% of all liver lesions found by ultrasound are benign [5]. Therefore, a fundamental knowledge of the prevalence and image morphology of hepatic hemangiomas, hepatic cysts, focal nodular hyperplasia (FNH), hepatic adenoma, and focal fatty sparing is essential. The number of recent ultrasound studies on the prevalence of benign liver lesions is relatively limited. In the past 10 years, comparatively few ultrasound-based studies have appeared on this subject [6–9]. Comparison of the study results is also difficult, because the studies differ with regard to the selection of the population investigated, the number of individuals investigated, and the diagnostic method used [ultrasound (US), computed tomography (CT), magnetic resonance imaging (MRI) or autopsy]. This is also ultimately reflected in the prevalence rates determined in the respective studies. Published values for the prevalence of hepatic hemangiomas range from 0.1% to 20.0% [6, 7, 10, 11] and those for hepatic cysts from 0.06% to 17.8% [7, 9, 10]. Only a few studies have determined the prevalence of FNH, hepatic adenoma and focal fatty sparing. The prevalence of FNH lies between 0.8% and 3.2% [12–15], of hepatic adenoma from 0.4% to 1.5% [11, 12, 15, 16], and of focal fatty sparing between 7.2% and 19.8% [8, 17, 18]. Several studies on the prevalence of focal liver lesions are primarily concerned with just one individual type of lesion or they investigate the prevalence for a given pre-existing disease [11, 19–21]. In addition, many studies on the prevalence of benign space-occupying lesions of the liver were conducted in the 1990’s or earlier, i.e., at a time when the image quality of the ultrasound devices used was greatly inferior [17, 21–23]. Without continually carrying out new studies at regular intervals, no comparative statements concerning possible changes in the prevalence of benign focal liver lesions over time are possible.

The aim of this study was to evaluate the prevalence of hepatic hemangioma, FNH, hepatic cysts, focal fatty sparing, and hepatic adenoma in a large population of university hospital patients and to compare this with the values published in the literature.

## Methods

**Fig. 1:**
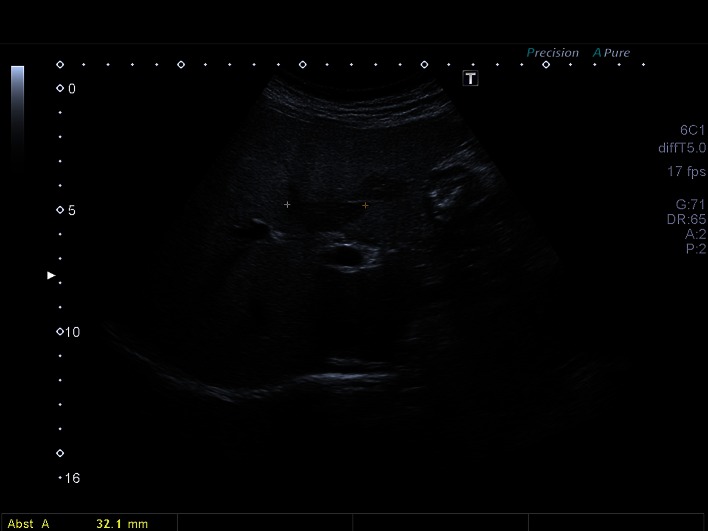
Focal fatty sparing

**Fig. 2:**
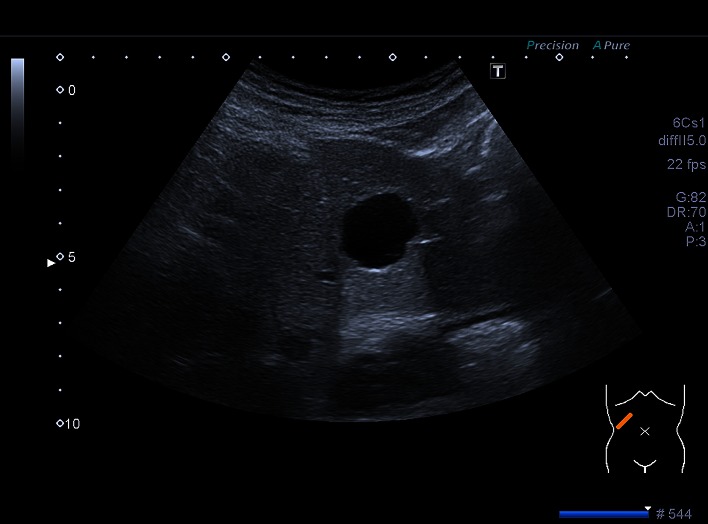
Cyst

**Fig. 3:**
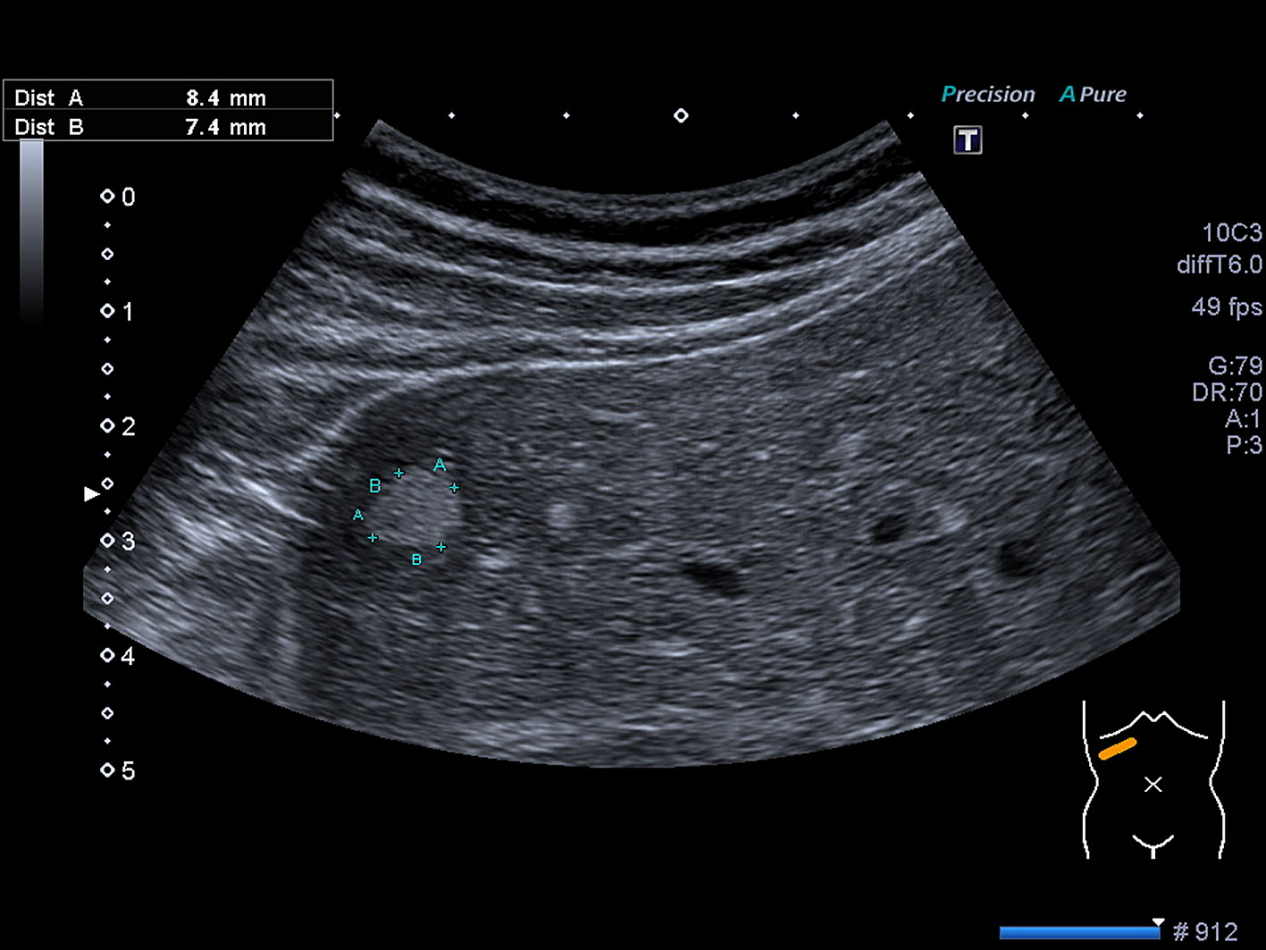
Hemangioma

**Fig. 4:**
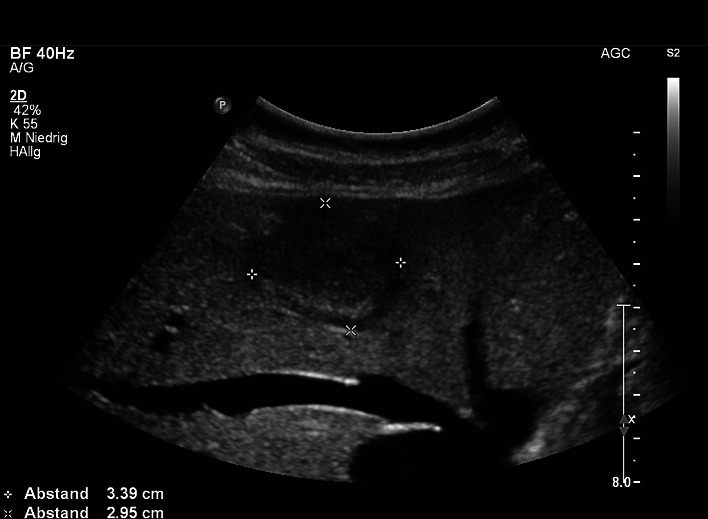
Focal nodular hyperplasia

**Fig. 5:**
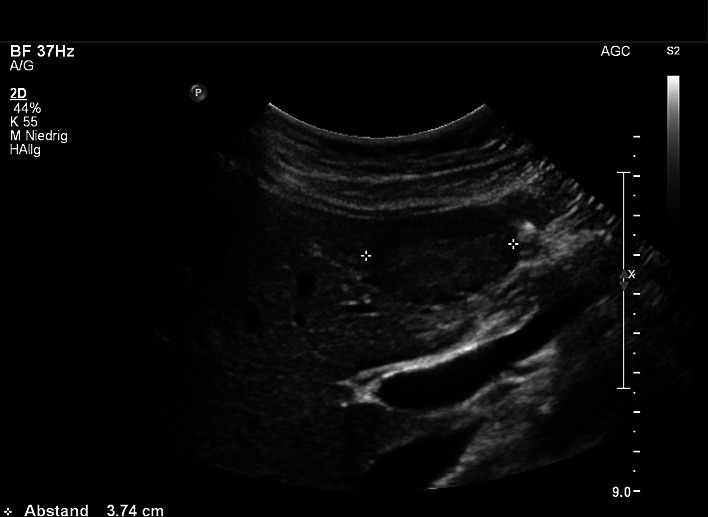
Adenoma

Data of 45,319 patients (48.5% women and 51.48% men) were analyzed using a PC-based, standardized documentation system (ViewPoint GE Healthcare GmbH Wessling/Oberpfaffenhofen, Germany). Patients had consecutively presented from January 2003 to November 2013 and had undergone abdominal ultrasound for a variety of diseases or for preventive medical examination in the university hospital. Inclusion criterion for this analysis was a positive sonographic diagnosis of benign focal liver lesions (hepatic cysts, hepatic hemangioma, FNH, hepatic adenoma and focal fatty sparing; Figs. 1, 2, 3,4 and 5). Results with inadequate or incomprehensible written or visual documentation of the finding were excluded from the study. Patients with polycystic liver diseases were excluded from this study. Ultrasound results typical of adenomas and FNH were only included in the evaluation, if these had been confirmed by further imaging or histology. The diagnosis of FNH was confirmed primarily by CEUS. At further existing unclarity, an MRI was performed in unclear findings at MRI puncture of the lesions were attempted.

All ultrasonographic examinations were performed in the ultrasound unit by experienced physicians. Examinations were performed using following devices: Philips HDI 3000, HDI 5000, IU22, Toshiba Aplio 500, and Siemens Acuson S3000. The used probes are C2-5, C1-5, and C1-6 (1–6 MHz). All lesions were examined also by color and power Doppler ultrasound. The liver was examined in inter- and/or subcostal planes with a fan-like motion allowing assessment of both the hepatic parenchyma and the intrahepatic bile ducts.

Following parameters were recorded and evaluated on the basis of the above-mentioned research question: positive/negative finding, nature of tumor, age and gender of the patient, number of foci (solitary, multiple), the respective ultrasound characteristics of the focus and size of the tumor (maximum diameter). The number of focal lesions was considered separately up to a figure of five lesions. From six or more foci upwards, these were combined as “more than five lesions.” The information about lesion size was based on the maximum measurable diameter in each case. If no unequivocal and/or acceptable statements about the above-mentioned parameters could be made from the re-inspected ultrasound images, these were expanded and/or measured again. Used criteria for the diagnosis of the lesions are presented in Table 1.

**Table 1:** Criteria Used to Diagnose Each Lesion Type

Liver lesion	Criteria
Focal fatty sparing	*Localization*
	Adjacent to the porta hepatis (segment IV)
	Gallbladder fossa
	Adjacent to the falciform ligament
	*B*-*mode presentation*
	Fatty liver
	Hypoechoic lesion
	Sharp edge
Hemangioma	Hyperechoic
	Sharp edge
	Round or oval form
	No blood flow in Doppler and Power Doppler-Mode
Cyst	Echo empty
	Posterior acoustic enhancement
	Border shadow sign
	Hyperechoic outlet echo
FNH	Spoke-wheel distribution in Doppler and Power-Doppler
	Central feeding arterial vessel
	Inhomogeneous
Adenoma	Hypoechoic/isoechoic
	Difficult to define

The study was conducted in accordance with the Guidelines of the Declaration of Helsinki and the recommendations of Good Clinical Practice. The project received a positive opinion from the local ethics committee (No. 377/13).

The statistical calculations were carried out using the statistics software SAS 9.2 (SAS Institute Inc., Cary, North Carolina, USA) and the data evaluated using descriptive statistics. For continuous variables, the mean and standard deviation were calculated, while categorical attributes were presented in absolute and relative frequencies. In order to demonstrate differences between patients with and without a lesion, the Wilcoxon signed rank-sum test was applied for continuous variables and the *χ*^2^ test for categorical variables, unless the sample size was too small, in which case Fisher’s exact test was used. The level of significance was set at *α* = 5%, and the p value was given to four decimal places.

## Results

Between 01/2003 and 11/2013, the liver was examined by ultrasound in a total of 45,319 patients, of whom 48.5% were women (*n* = 21,988) and 51.48% men (*n* = 23 331). The mean age of patients at the time of the investigation was 56 ± 18.1 years (range: 4 months–105 years). On average, women were aged 56.1 ± 18.8 years and men 55.9 ± 17.4 years. At least one of the lesions of interest was found in a total of 6851 patients (15.1% of the population examined). A total of 44.9% of these patients were outpatients and 55.1% inpatients. The most common lesion was focal fatty sparing, which was diagnosed in 2839 cases, corresponding to a prevalence of 6.3%. It was most often found in the 51–60 years age group; the mean age of the patients with focal fatty sparing was 54.9 ± 14.5 years. Age-specific prevalence was far less apparent in the younger age groups and in the elderly. Men were affected much more often (63.5%) than women (36.5%). The mean maximum measured size of the focal fatty sparing was 20.6 mm. All the cases of focal fatty sparing that we encountered were solitary findings in its typical location in liver segment IV in the region of the gallbladder bed. The age-dependent frequency of hepatic steatosis and the related prevalence of focal fatty sparing in patients with hepatic steatosis were also determined (Table 2). The frequency of focal fatty sparing in patients with hepatic steatosis decreased with age and in the youngest age group of patients with hepatic steatosis, namely under 30 years of age, the specific prevalence of focal fatty sparing was over 90%. In our study, this figure fell almost linearly with increasing age and amounted to only 66.8% in the group of patients over 70 years of age.

**Table 2:** Age-Specific Prevalence of Focal Fatty Sparing in Hepatic Steatosis

	Hepatic steatosis	Focal fatty sparing	Prevalence of focal fatty sparing (%)
Age classes			
<30	164	154	93.90
31–40	298	272	91.28
41–50	629	545	86.64
51–60	899	718	79.87
61–70	920	685	74.46
>70	696	465	66.81
Gender			
Male	2165	1802	83.23
Female	1441	1037	71.96

The second most commonly diagnosed liver lesion was the hepatic cyst, with 5.8% (*n* = 2631). Hepatic cysts became more common with age. The youngest patient group, under 30 years of age, was scarcely affected, with an age-specific prevalence of 0.8% (*n* = 21). Most hepatic cysts were found in the oldest patients, with a frequency of 38.5% (*n* = 1012). The mean age was 64.7 years. Women were somewhat more affected (56.1%, *n* = 1477) than men (43.9%, *n* = 1154). The largest measured cyst diameter averaged 22.3 mm. Solitary cysts were found in 62.8% (*n* = 1652) of cases.

In our patient population, the prevalence of hepatic hemangioma was 3.6% (*n* = 1640). As regards the age distribution, the respective age-specific prevalence began with 7.0% (*n* = 115) in the youngest patients and rose to a maximum of 22.5% (*n* = 369) of all discovered hemangiomas in the age group between 51 and 60 years. Lower prevalence was again determined in the highest age groups. The gender distribution of hemangioma was almost balanced, with 53.4% (*n* = 879) women/46.6% (*n* = 761) men. 76.67% (*n* = 1157) of diagnosed hemangiomas were solitary, and the average size of the hemangiomas was 20.1 mm.

The prevalence of FNH was 0.2% (*n* = 81). The highest prevalence was found in younger women, and 86.4% (*n* = 70) of all patients with FNH were females. The peak age for FNH occurred in the youngest patient group with 34.6% (*n* = 28) of the diagnosed lesions and fell continuously with increasing age. Multiple FNHs were rare; the prevalence of solitary FNHs was 88.9% (*n* = 72), and the average size was 51.6 mm.

With only 19 cases and hence a prevalence of 0.04%, hepatic adenoma was the rarest of the liver lesions we investigated. Overall, more adenomas were diagnosed in the younger patient groups under 50 years of age than in the older ones. In our population, a maximum occurred at between 41 and 50 years of age. 84.2% (*n* = 16) of patients with adenoma were women. Apart from 2 exceptions, all diagnosed adenomas were solitary findings (89.5%, *n* = 17). The mean size was 39.0 mm (Table 3).

**Table 3:** Results on the Prevalence and Epidemiological Distribution of Benign Liver Lesions

	Focal fatty sparing	Hemangioma	Cyst	FNH	Adenoma
	N (%) Mean ± SD				
Number	2839 (6.26%)	1640 (3.62%)	2631 (5.81%)	81 (0.18%)	19 (0.04%)
Age (years)	54.87 ± 14.53	52.61 ± 15.10	64.70 ± 13.03	37.83 ± 14.20	40.68 ± 11.60
Age groups					
<30	154 (5.42%)	115 (7.01%)	21 (0.80%)	28 (34.57%)	4 (21.05%)
31–40	272 (9.58%)	226 (13.78%)	62 (2.36%)	24 (29.63%)	4 (21.05%)
41–50	545 (19.20%)	355 (21.65%)	255 (9.69%)	15 (18.52%)	7 (36.84%)
51–60	718 (25.29%)	369 (22.50%)	534 (20.30%)	7 (8.64%)	2 (10.53%)
61–70	685 (24.13%)	323 (19.70%)	747 (28.39%)	3 (3.70%)	2 10.53%)
>70	465 (16.38%)	252 (15.37%)	1012 (38.46%)	4 (4.94%)	–
Gender					
Male	1802 (63.47%)	761 (46.40%)	1154 (43.86%)	11 (13.58%)	3 (15.79%)
Female	1037 (36.53%)	879 (53.60%)	1477 (56.14%)	70 (86.42%)	16 (84.21%)
Number of lesions					
1	2839 (100%)	1157 (76.67%)	1652 (62.79%)	72 (88.89%)	17 (89.47%)
2	–	219 (14.51%)	419 (15.93%)	4 (4.94%)	2 (10.53%)
3	–	72 (4.77%)	198 (7.53%)	3 (3.70%)	–
4	–	21 (1.39%)	111 (4.22%)	1 (1.23%)	–
5	–	16 (1.06%)	44 (1.67%)	1 (1.23%)	–
>5	–	24 (1.59%)	207 (7.87%)	–	–
Size (mm)	20.56 ± 10.23	20.06 ± 15.05	22.28 ± 19.06	51.63 ± 29.47	38.95 ± 27.47

## Discussion

Studies concerning the prevalence of benign focal liver lesions present a quite heterogeneous picture as regards the precise research question posed, the size of the population studied, and the investigative methods used. It is therefore difficult to compare the various study results and apply them to routine ultrasound primary diagnostics. For example, the prevalence of hepatic hemangioma determined in the studies ranged from 0.1% to 20.0% and that of hepatic cysts from 0.06% to 17.8%. Only a very few studies investigated the prevalence of FNH, hepatic adenoma, and focal fatty sparing. The prevalence figures reported in previous ultrasound studies for FNH were 0.8%–3.2% and for hepatic adenoma 0.4%–1.5% [8, 15, 17, 20].

Liver areas with reduced focal, rarely zonal accumulation of fat can occur in hepatic steatosis. The most common site of these pseudo lesions are hepatic segments IV and V, the gallbladder bed, the falciform ligament region, and ventral to the portal vein. In rare cases, focal fatty sparing has also been described in other liver segments where, in the first instance, it is generally difficult to distinguish from malignant lesions and can hence pose considerable problems for a differential diagnosis [24–26]. To date, only a few studies have been published on the prevalence of focal fatty sparing or of focal fat distribution disorders in the liver [8, 27]. Kratzer et al. calculated a value of 9.05% in a random population collective [8]. The research group of Koseoglu reported a prevalence of focal fatty sparing of up to 19.8 [18] Strunk et al. reported a prevalence of 7.2% in a population of patients with colorectal carcinoma [17]. Our result of 6.3% is below the prevalence figures of Kratzer et al. and Strunk et al. [8, 17]. The size and age structure of the respective study populations, as well as the quality of the ultrasound equipment used, need to be considered here. As also found by Aubin et al., one possible cause could be the lower clustering of focal fatty sparing in patients with status post cholecystectomy, whose number increases with age and occurs more frequently in a hospital population than in a random sample of the entire population [24].

Considerably more studies have investigated the prevalence of hepatic hemangioma than of focal fatty sparing, FNH, and adenoma [6, 7, 9, 11, 21]. The majority of the more recent ultrasound-based prevalence studies show significantly higher prevalence figures than older ultrasound-based studies (Table 4). Our prevalence figure of 3.6% for hemangioma lies in the mid-range compared to the previously published results from ultrasound-based studies [6, 7, 9, 17]. In comparison with CT, MRI, and autopsy studies, which show a far higher range of prevalence, our figure is in the lower third [10, 11, 19, 20]. The highest prevalence figures were reported from autopsy and CT studies [12, 15, 19, 28]. In terms of the age distribution and average size, our results correspond to those of comparable studies [13, 19]. Gandolfi et al. described a higher prevalence of hemangioma in middle aged or older patients, while Rungsinaporn et al. reported a higher prevalence of hepatic hemangioma in women—resulting that we were unable to corroborate with our data (Table 3) [21, 29].

**Table 4:** Ultrasound Studies on the Prevalence of Focal Liver Lesions

Study	Population	P/R	Hemangioma	FNH	Cysts	Adenoma	FFS
			%				
Massironi et al. [6]	Patient population (*n* = 1449)	R	5.7	–	–	–	–
Khosa et al. [7]	Patient population (*n* = 1008)	R	0.3	–	1.4	–	–
Kratzer et al. [8]	Normal population (*n* = 1624)	R	–	–	–	–	9.1
Varbobitis et al. [9]	Normal population (*n* = 47,045)	R	0.45	–	1.3	–	–
Rungsinaporn et al. [29]	Normal population (*n* = 3398)	R	3.6	–	8.7	–	–
Linhart et al. [13]	Patient population (*n* = 731)	P	5.7	0,8	10.2	–	–
Caremani et al. [22]	Patient population (*n* = 26 514)	R	–	–	4.7	–	–
Strunk et al. [17]	Patient population (n = 166 with colorectal carcinoma)	R	6.6	–	7.8	–	7.2
Gandolfi et al. [21]	Patient population (*n* = 21,280)	R	1.4	–	–	–	–
Gaines et al. [23]	Patient population (*n* = 1695)	R	–	–	2.5	–	–

P, prospective; R, retrospective; FNH, focal nodular hyperplasia; FFS, focal fatty sparing

As with hemangioma, there are a comparatively large number of prevalence studies for hepatic cysts, but they also differ in terms of study size, patient populations investigated, and diagnostic techniques used. Retrospective and prospective studies based on ultrasound have reported prevalence data for hepatic cysts of between 0.1% and 11.3% [7, 9, 23, 29]. With our determined prevalence of 5.8%, we are also here in the mid-range. In the case of hepatic cysts, the range of prevalence figures from CT, MRI, or autopsy studies is also much wider than that of the ultrasound-based studies [5, 10, 15, 19, 30]. In relation to the CT, MRI, and autopsy studies, our prevalence is in the mid to lower third of the range. CT-supported studies have reported the highest figures for prevalence [15, 19]. In line with our results, all studies reported a higher prevalence of hepatic cysts with increasing age [19, 22, 23, 30]. Most studies have also found a gender-dependent aspect, with higher prevalence figures for hepatic cysts in women [22, 23, 29, 30]. Our measured mean cyst size of 2.2 cm corresponds to the values published in the literature [19, 22, 23, 30].

There are only a few studies on the prevalence of FNH [12–15]. The prevalence figures of the two ultrasound-based studies for FNH were 0.8% and 1.8% and lie below the figures of between 1.8% and 3.2% reported from CT, MRI, or pathological investigations [12–15]. Our figure of 0.18% is markedly lower than the data published to date. This difference can be related to the population size and age of the patients studied [13, 14]. Through higher performance ultrasound equipment and the introduction of contrast-enhanced ultrasound, the diagnosis of FNH can nowadays be made with higher sensitivity and specificity [31].

There are no ultrasound studies on the prevalence of hepatic adenoma within a large patient population. Furthermore, there are only a few prevalence studies based on CT, MRI investigations, and autopsy studies [11, 12, 15, 16]. The prevalence data published so far on hepatic adenoma are between 0.4% and 1.7%. The understanding of hepatic adenoma has changed fundamentally in recent years [32, 33]. Through the division into four different genotypic subtypes, new aspects have emerged concerning prevalence and clinical presentation. Naturally, these cannot be recorded in retrospective ultrasound prevalence studies. The prevalence of 0.04% determined by us is considerably lower than that found in the previous studies. As was already put forward as a possible explanation in the case of FNH, this could be due to the age of the study participants, the size of the patient population investigated, and the improved differentiation possibilities of modern ultrasound equipment. Unfortunately, we cannot compare our results on age and gender distribution or those concerning the average size of the hepatic adenoma with any of the studies available to us. However, the occurrence of a hepatic adenoma is associated with the ingestion of oral contraceptives, which may be reflected in our results, since we found the majority of adenomas in women aged below 50 years [34].

## Conclusions

In summary, our results show that the first possibility to be considered on the incidental discovery of space-occupying lesions of the liver—especially if hepatic steatosis is present—is focal fatty sparing. Simple hepatic cysts and hemangiomas are the most common focal liver lesions. The finding of a FNH or an adenoma is rarely a fortuitous result.
